# Functional and transcriptional characterization of complex neuronal co-cultures

**DOI:** 10.1038/s41598-020-67691-2

**Published:** 2020-07-03

**Authors:** Heather A. Enright, Doris Lam, Aimy Sebastian, Ana Paula Sales, Jose Cadena, Nicholas R. Hum, Joanne J. Osburn, Sandra K. G. Peters, Bryan Petkus, David A. Soscia, Kristen S. Kulp, Gabriela G. Loots, Elizabeth K. Wheeler, Nicholas O. Fischer

**Affiliations:** 1 Physical and Life Sciences Directorate, Lawrence Livermore National Laboratory, Livermore, CA USA; 2 Engineering Directorate, Lawrence Livermore National Laboratory, Livermore, CA USA; 3 University of California, Merced, School of Natural Sciences, Merced, CA USA

**Keywords:** Cellular neuroscience, Neuronal physiology

## Abstract

Brain-on-a-chip systems are designed to simulate brain activity using traditional in vitro cell culture on an engineered platform. It is a noninvasive tool to screen new drugs, evaluate toxicants, and elucidate disease mechanisms. However, successful recapitulation of brain function on these systems is dependent on the complexity of the cell culture. In this study, we increased cellular complexity of traditional (simple) neuronal cultures by co-culturing with astrocytes and oligodendrocyte precursor cells (complex culture). We evaluated and compared neuronal activity (e.g., network formation and maturation), cellular composition in long-term culture, and the transcriptome of the two cultures. Compared to simple cultures, neurons from complex co-cultures exhibited earlier synapse and network development and maturation, which was supported by localized synaptophysin expression, up-regulation of genes involved in mature neuronal processes, and synchronized neural network activity. Also, mature oligodendrocytes and reactive astrocytes were only detected in complex cultures upon transcriptomic analysis of age-matched cultures. Functionally, the GABA antagonist bicuculline had a greater influence on bursting activity in complex versus simple cultures. Collectively, the cellular complexity of brain-on-a-chip systems intrinsically develops cell type-specific phenotypes relevant to the brain while accelerating the maturation of neuronal networks, important features underdeveloped in traditional cultures.

## Introduction

In vitro brain-on-a-chip platforms have emerged as useful tools to model brain activity to aid in evaluating neuronal outcomes for new drugs and toxicants, in addition to elucidating disease mechanisms^1–3^. These in vitro approaches often utilize multi-electrode arrays (MEA), which allow for non-invasive interrogation of in vitro neuronal networks formed de novo from dissociated rodent or human neurons or from networks established in rodent brain tissue slices^4–7^. The use of dissociated neurons offers an amenable approach for establishing and evaluating human-relevant responses using human primary or stem-cell derived neurons and glial cell types^7–11^, since human brain slices are not often available. Brain-on-a-chip efforts incorporating either rodent or human cell types have been used for toxicology screening^12–14^, developing integrated systems (i.e. neurovascular units comprised of a blood–brain barrier and brain parenchyma^3, 15^, and creating more relevant architectures using three-dimensional cultures^16–18^. In addition, engineered platforms have been designed to enable controlled placement of neurons (e.g. cortical, hippocampal, amygdala) to characterize region-specific networks^19, 20^, or to isolate axons (or axonal bundles) for analysis^21, 22^. Electrophysiological features of rodent-derived neural networks, established with both glutamatergic and GABAergic neurons, have been well characterized using dissociated neurons from primary cells or derived from neural stem cells^23–25^. However, these systems most often utilize mono-cultures of neurons or co-cultures with astrocytes, which do not fully mimic the cellular complexity of the brain and may ultimately misrepresent neuronal responses when evaluating drugs and toxicants relative to in vivo testing^26^.

To more fully model brain heterogeneity, we have developed a complex in vitro system wherein rat cortical neurons are co-cultured with glial cell types (astrocytes and oligodendrocytes) at ratios relevant to the postnatal brain^27–29^ and have evaluated morphological, molecular, and functional differences of this complex system relative to a simpler neuronal culture over 31 days in vitro (DIV). Immunostaining was used to compare the cellular composition and structure and single cell RNA sequencing was used to identify molecular changes between the two culture systems. To elucidate whether the neurophysiological responsiveness (i.e. network activity) is influenced by cellular composition and structure (e.g., alignment of MBP to mature axons), we challenged both simple and complex cultures to bicuculline, a gamma-aminobutyric acid A (GABA_A_) receptor antagonist commonly used to evaluate the disinhibition of GABAergic neural networks in vitro.

Increasing the cellular and structural complexity of the complex neuronal in vitro system accelerated and enhanced de novo network maturation. This was supported by distinct morphological and functional (electrophysiology) changes in the co-culture system. Our study underscores the importance of increasing the complexity of neuronal cultures to aid in improving the functional relevance of brain-on-a-chip systems to better mimic the in vivo brain*.* We anticipate these findings will extend to human-based neuronal cultures and will more accurately reproduce the drug responses observed in in vivo systems.

## Results

### Culture characterization

In vitro neuronal cultures for both simple (neurons and low levels of contaminating astrocytes, oligodendrocytes) and complex systems (neuronal cultures supplemented with defined ratios of astrocytes and oligodendrocytes) were established on MEAs and routinely monitored using electrophysiology over the course of 31 days . The seeding composition of the complex system was determined using published in vivo ratios of neurons and glial cell types during the rodent postnatal period^27–29^. As such, complex cultures were seeded with ~ 79% neurons, ~ 16% astrocytes and ~ 5% oligodendrocyte precursor cells.

**Figure 1:**
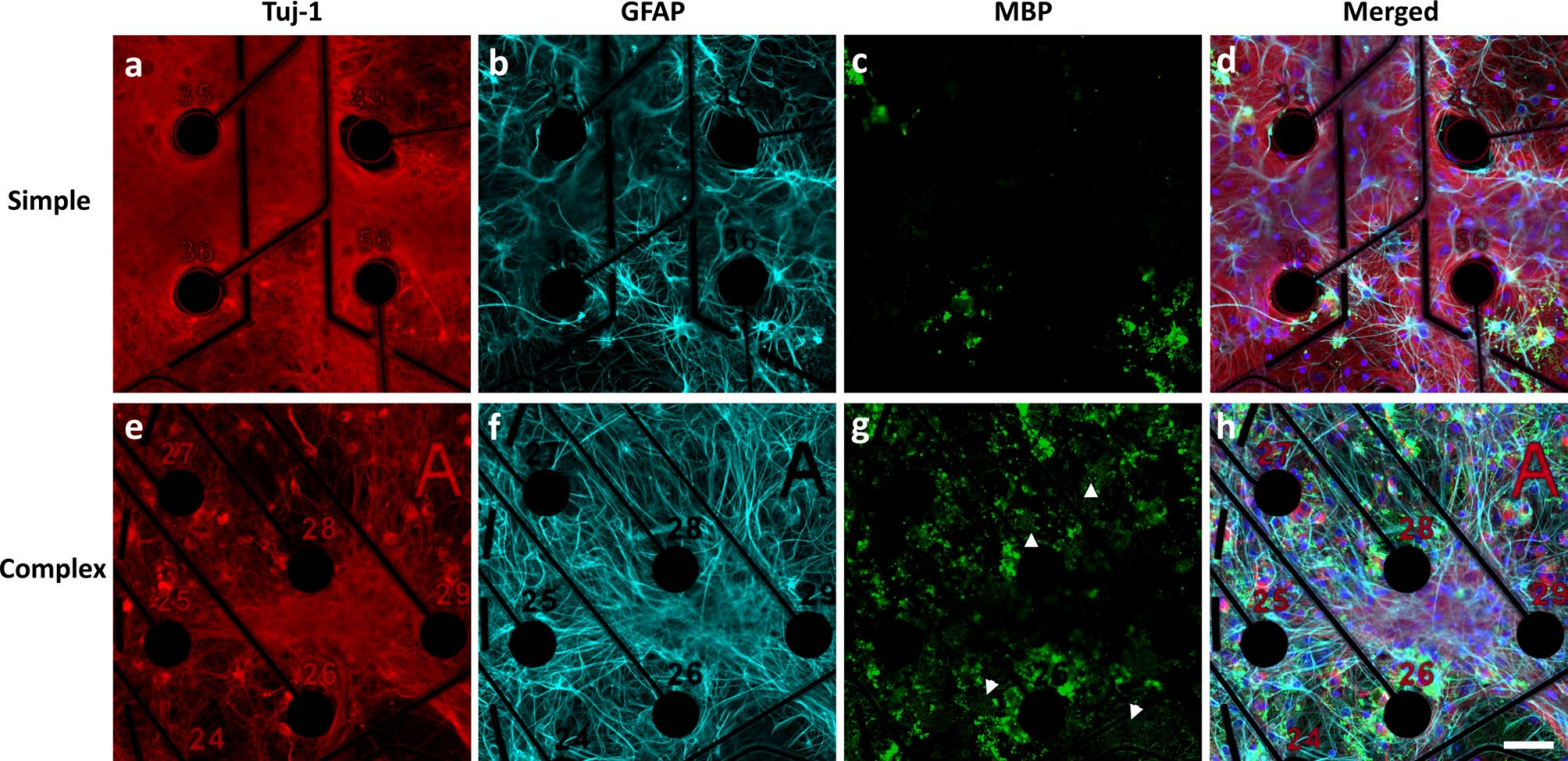
Immunofluorescence characterization of cortical cultures in simple and complex systems at DIV31. Neurons were identified by staining for Tuj-1 (Neuron-specific class III beta-tubulin, **a**, **e**). Glial fibrillary acidic protein (GFAP) was used to identify astrocytes (**b**, **f**) and myelin basic protein (MBP) was used to identify mature oligodendrocytes and myelin (white arrowheads) (**c**, **g**). Merged images with nuclear stain (DAPI, blue) are shown in (**d**) and (**h**). Figure has been modified to remove electrode autofluorescence. Scale bar = 50 µm.

At the end of the study (DIV31)**,** simple and complex cultures were characterized using immunocytochemistry to quantify neuronal density, identify specific cell types and to evaluate cell and network morphology (Fig. 1, Supplementary Figs. S1–S4). Neurons and astrocytes were identified using antibodies against neuron-specific class III beta-tubulin (tuj-1) and glial fibrillary acidic protein (GFAP), respectively^30–32^. To verify the maturation of oligodendrocyte precursor cells into mature oligodendrocytes, the cultures were probed with an antibody against myelin basic protein (MBP), a protein produced solely by mature oligodendrocytes^33–35^. Differences in cell morphology, cell-type distribution, and cell-specific biomarker localization were observed between the two different cultures. For example, while neuronal seeding densities were identical, neurons in the complex system were more localized in dense regions (Fig. 1e) compared to the diffuse distribution observed in the simple system (Fig. 1a). In addition, finer neuronal processes were also noted in simple cultures (qualitative observation). Quantification of neuronal cell counts between simple and complex conditions (Supplementary Fig. S2) showed slightly higher levels of neurons within simple (32.10% ± 0.02) compared to complex cultures (25.00% ± 0.02, p < 0.01). We also stained for synaptophysin, a known pre-synaptic marker, to evaluate synaptic expression between systems. Synaptophysin was localized to tuj-1-expressing cells in both culture systems (Supplementary Fig. S3). Compared to simple cultures where distribution was more widespread, complex systems showed synaptophysin expression localized in distinct areas. GFAP-positive astrocytes in the co-cultures appeared more branched and stellate-like with rounded somas (Fig. 1f) when compared to the flatter morphology observed in the simple system, which contained a lower level of astrocytes due to astrocyte contamination in the neuron cell stock (Fig. 1b). A higher density of MBP was noted in the complex system, which further exhibited defined areas of myelin production (white arrow heads, Fig. 1g). Additionally, the complex system appeared to arrange in multiple cell layers (Supplementary Fig. S4) spanning at least 25 μm. In contrast, the simple cultures were organized in a planar monolayer (data not shown).

**Figure 2:**
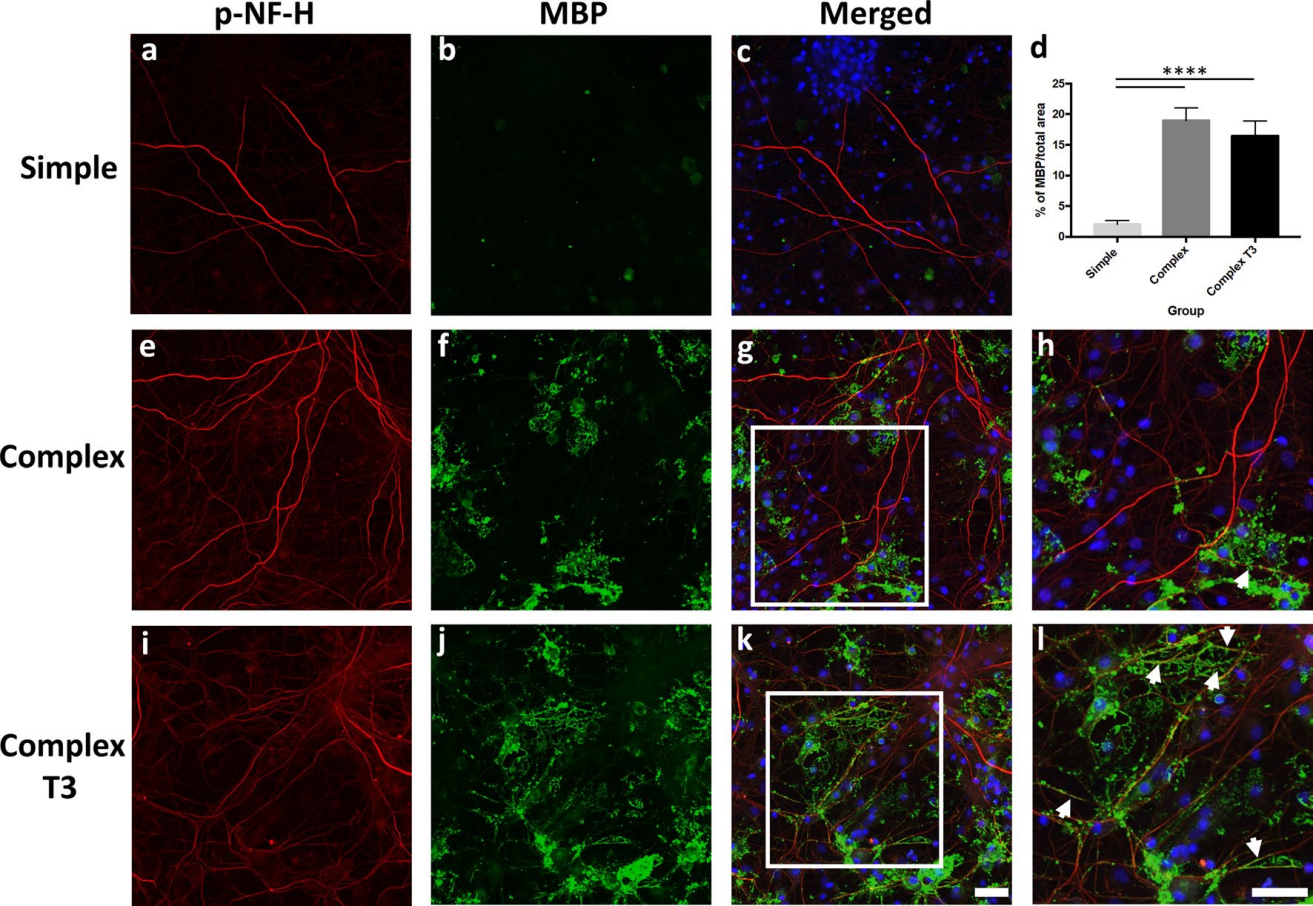
Immunofluorescence characterization of myelination in simple and complex systems at DIV31. Mature axons were identified by staining for p-NF-H (phosphorylated neurofilament H, **a, e, i**). Myelin basic protein (MBP) was used to identify mature oligodendrocytes and myelin (**b, f, j**). Merged images with nuclear stain (DAPI, blue) are shown in (**c**), (**g**) and (**k**). Zoomed-in images of MBP/axon co-localization (white boxes) from panels (**g**) and (**k**) are shown in (**h**) and (**l**), respectively. White arrowheads indicate areas of co-localization. Quantification of MBP in both simple and complex systems is shown in (**d**). Scale bar = 50 µm. ****p < 0.0001.

Cells in the complex cultures were maintained in neuronal media supplemented with (complex T3) and without (complex) triiodothyronine (T3). T3 has been shown to enhance myelination of axons in both CNS and spinal culture systems^36^. We evaluated both systems (complex T3 and complex) to determine if T3 elicited morphological (i.e. co-localization of myelin with axons) and functional (electrophysiology) changes. Cells were immunostained with MBP and a mature axonal marker (phosphorylated neurofilament-H, p-NF-H). Maturation from oligodendrocyte precursor cells to oligodendrocytes occurred in both complex systems, and the presence of mature oligodendrocytes (MBP-positive cells) and myelin was noted (Fig. 2f, j). MBP staining with respect to mature axons (p-NF-H) is shown for simple, complex, and complex T3 groups in Fig. 2. Significantly greater MBP expression was observed between the complex systems and simple culture (Fig. 2d, p < 0.0001). However, no significant difference in total MBP expression was observed between the two complex culturing conditions (Fig. 2d), although differences were observed in the co-localization of MBP with mature axons in the complex T3 system. For the complex system without T3, substantial MBP+ staining was observed, but co-localization with p-NF-H was not widespread (Fig. 2g, h). A greater degree of co-localization was noted for the complex T3 group in regions of the culture where mature oligodendrocytes were also present (Fig. 2h, l, white arrowheads). Clear areas of myelin were noted near MBP+ cells, with defined areas of MBP/p-NF-H overlap (Fig. 2k, l).

**Figure 3:**
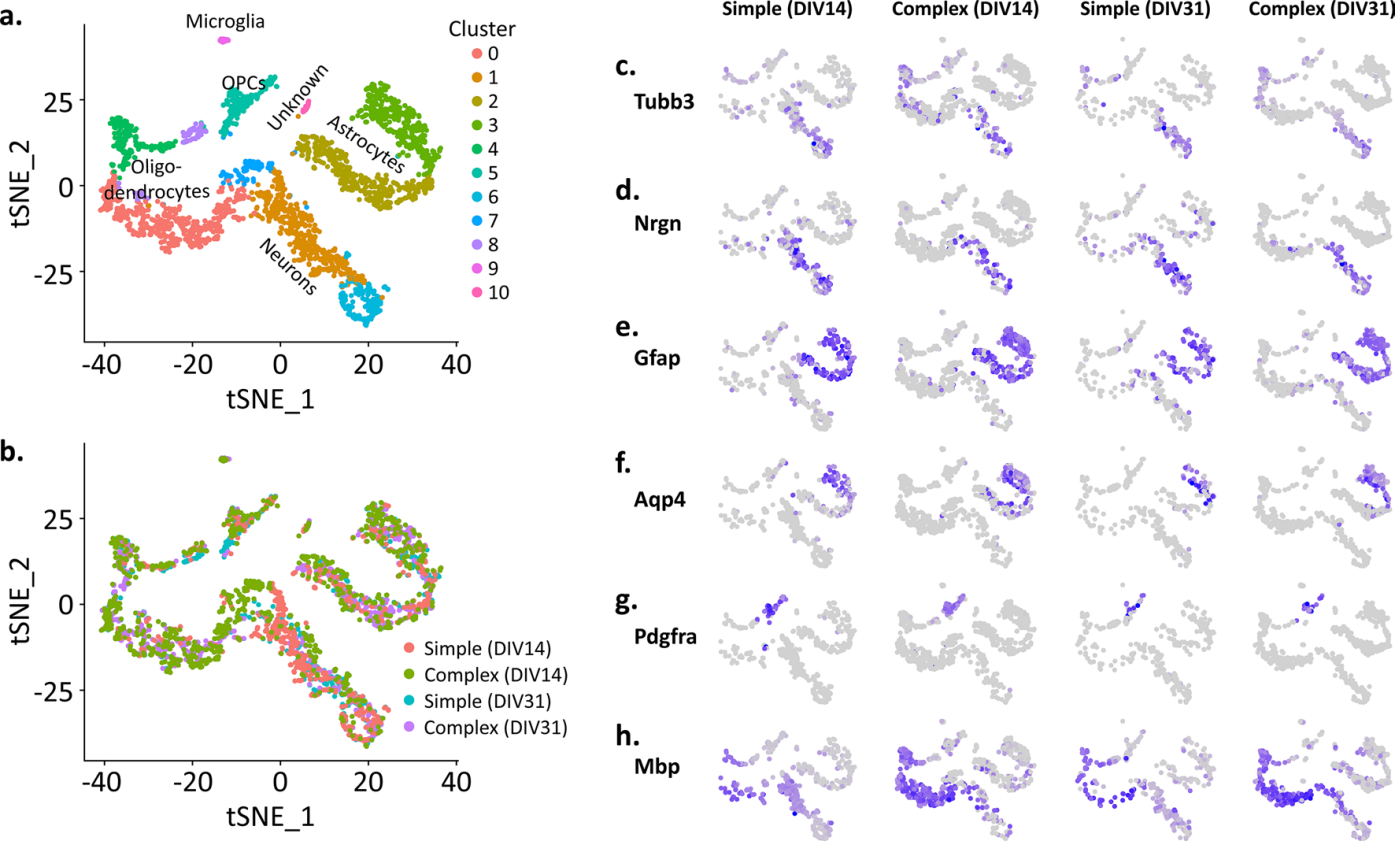
Single-cell RNAseq data. (**a**) t-SNE plot showing eleven different cell clusters. Different cell types (and sub-types) are color-coded. (**b**) t-SNE plot of all cells for both simple and complex groups at each DIV. Gene markers for specific cell types are shown in (**c**–**h**); *Tubb3* and *Nrgn* for neurons (**c**, **d**); *Gfap* and *Aqp4* for astrocytes (**e**, **f**); *Pdgfra* and *Mbp* for oligodendrocytes (**g**, **h**).

In addition to immunostaining, simple and complex cultures were examined using single cell RNA sequencing (scRNAseq) at DIV14 and 31 to characterize cellular heterogeneity over time as well as to identify cellular processes that were distinct between simple and complex cultures. Eleven cell clusters were detected (Fig. 3a), with multiple clusters present for each of the cell types seeded (neurons, astrocytes and oligodendrocytes) as well as a very low level of microglia (cluster 9). Cell clusters with respect to DIV are shown in Fig. 3b. Cell cluster identity was determined using a variety of established cell-type marker genes (Supplementary Fig. S5)^37, 38^. Feature plots for each culture system and time point (i.e. simple at DIV14) are shown for a select number of marker genes in Fig. 3c–h, and include *Tubb3* and *Nrgn* for neurons*, Gfap* and *Aqp4* for astrocytes, *Pdgfra* and *Mbp* for oligodendrocytes. *Ptprc* and *Csf1r* were used to classify microglia, but not enough cells were identified to generate feature plots for each culture system and time point*.* Similar to what was observed at the protein level using immunocytochemistry (Fig. 1), transcriptome analysis confirmed a low level of astrocytes and oligodendrocytes present within our simple cultures (Fig. 3e–h). For oligodendrocytes, this included low levels of both immature (*Pdgrfa*^+^) and mature (*Mbp*^+^) cell types.

**Figure 4:**
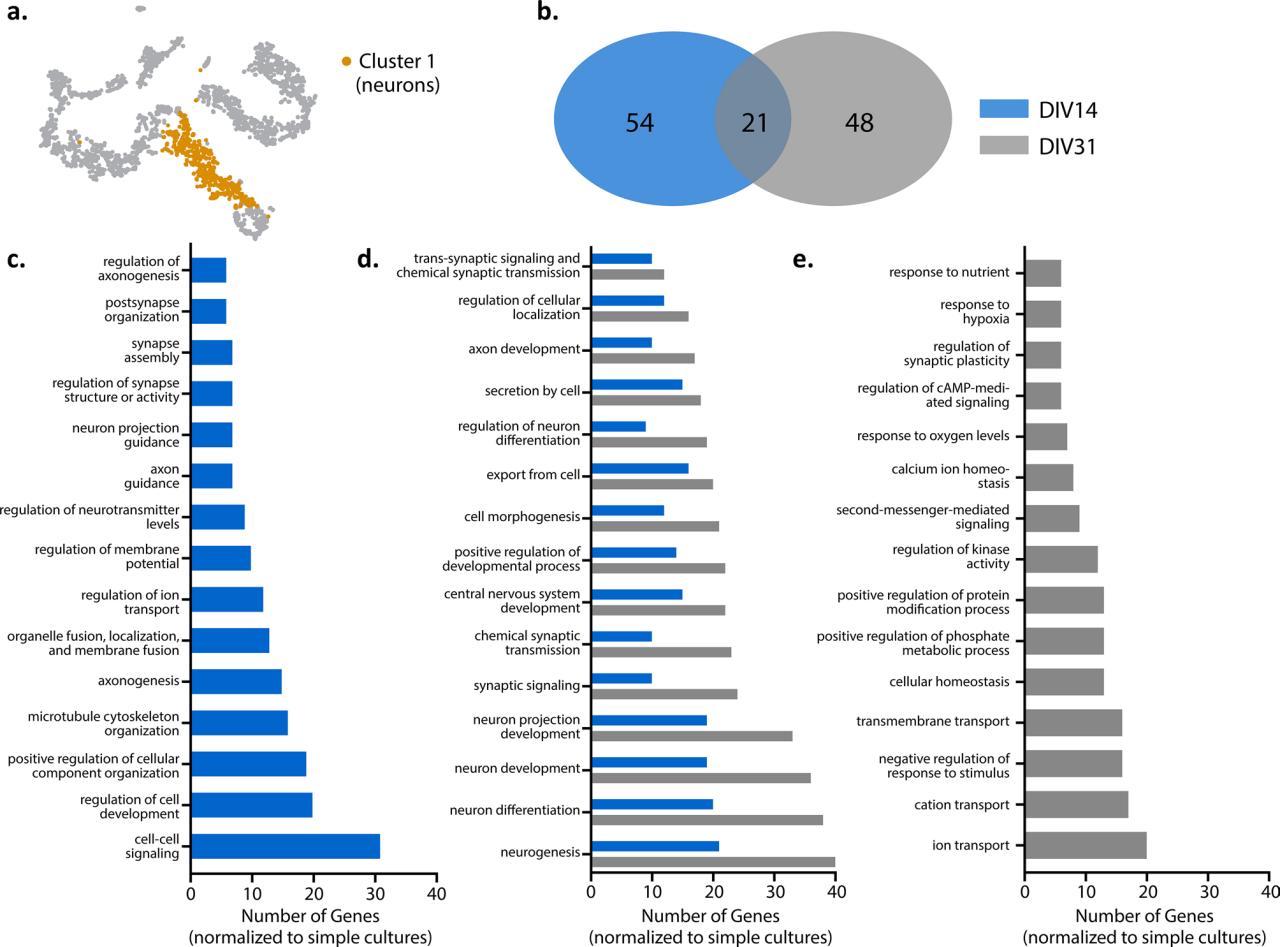
Gene ontology enrichment analysis of cluster 1 neurons at DIV14 and DIV31. (**a**) t-SNE plot illustrating cluster 1 neurons. (**b**) Venn diagram illustrates the number of distinct and similar biological processes at DIV14 and DIV31. Bar graphs (**c**–**e**) illustrates the top 10 biological processes that are distinct at DIV14 (**c**), common between time points (**d**) and distinct to DIV31 (**e**).

Next, we examined whether any cellular processes were transcriptionally different between our culturing conditions, in specific cell populations, over time. Neurons within our culture system formed two distinct clusters (i.e., cluster 1 and 6) (Fig. 4a). To understand the significance of these two neuronal subpopulations, we used gene ontology (GO) analyses to both identify biological processes associated with the genes differentially expressed (e.g., logFC > 0.25 or < − 0.25) between these neuron clusters and evaluate changes in these processes in our complex culture system over time relative to the simple culture (Fig. 4b–e). Neuron clusters 1 and 6 demonstrated an overlap in biological processes involved in neuronal network formation, organization, and signaling (Fig. 4d). Our main neuronal cluster, cluster 1 (Fig. 4c), exhibited higher expression for genes associated with biological processes for non-neuronal cells within our complex cultures, suggesting neuron-glia interaction (e.g. astrocyte differentiation, myelination, and ensheathment of neurons and axons). The key intracellular signaling cascade (ERK1/2), which plays a role in regulating the proliferation, differentiation, and survival of various cell types as well as neuroplasticity, was also noted^39, 40^. In cluster 6 (Fig. 4e), processes related to neuronal network and synapse formation were prevalent, suggesting that an immature neuronal subpopulation persisted in long-term cultures.

**Figure 5:**
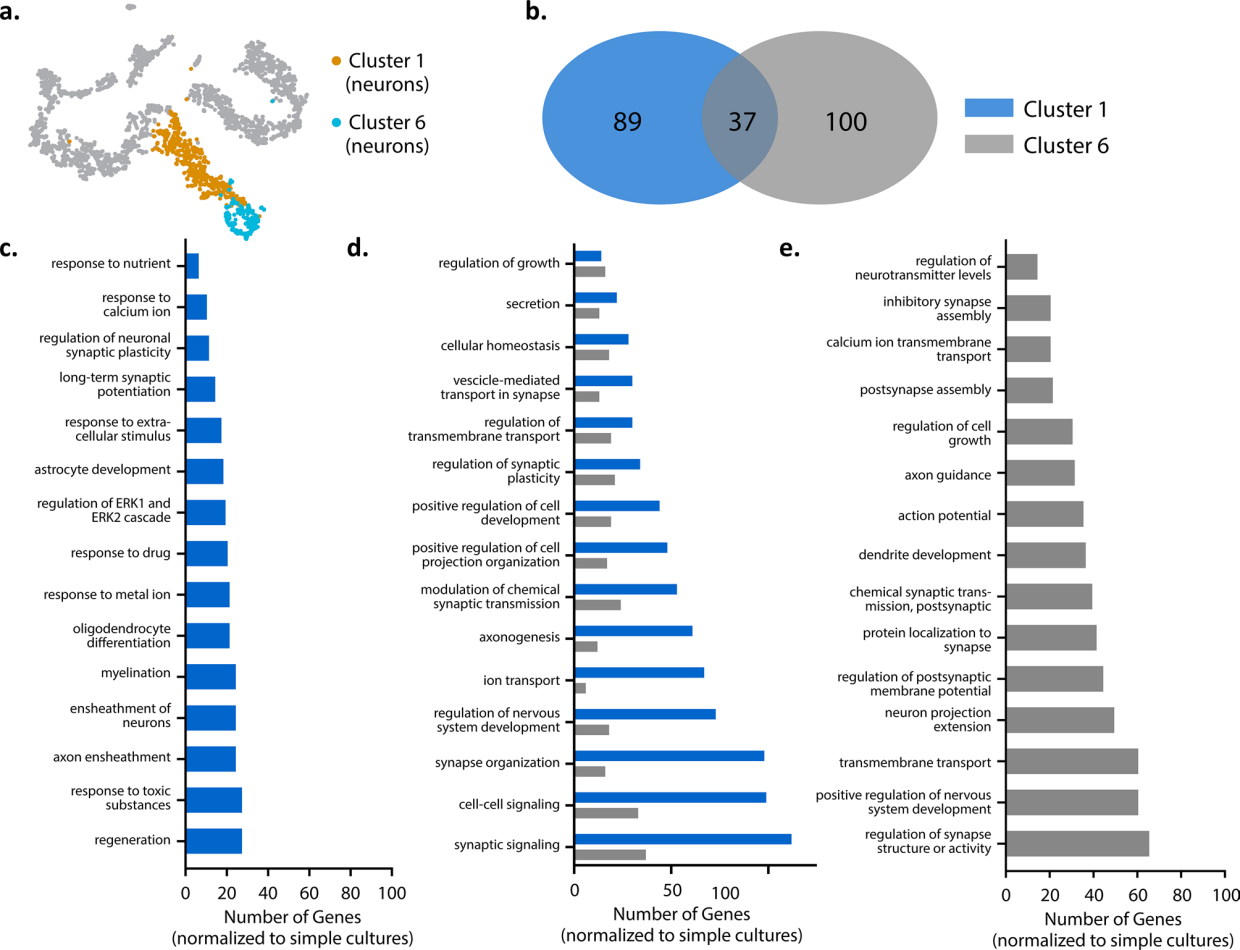
Gene ontology enrichment analysis of cluster 1 and 6 neurons at DIV31. (**a**) t-SNE plot illustrating cluster 1 and 6 neurons. (**b**) Venn diagram illustrating distinct and similar biological processes identified by GO between cluster 1 and 6. (**c**) Representative distinct processes from the 89 identified for cluster 1. (**d**) Representative similar processes from the 37 identified between cluster 1 and cluster 6 and (**e**) Representative distinct processes from the 100 identified from cluster 6.

Next, we compared gene expression at DIV14 and DIV31 to understand whether biological processes in cluster 1 evolved over time (Fig. 5). While there were common biological processes between the time points, there were also biological processes specific to each DIV (Fig. 5b). At DIV14 (Fig. 5c), key processes in our complex cultures related to neuronal network development, including regulation of various aspects of our neuronal networks (i.e. cell differentiation, neurotransmitters, synapse organization) as well as neuronal network and synapse formation (i.e. axon guidance, axonogenesis, cell–cell signaling, neuron projection guidance). At DIV31 (Fig. 5e), more biological processes involved in cell regulation and responsiveness were observed, such as intracellular transport (i.e. ion, cation, transmembrane), homeostasis, and responses to environment. We did note similarities between both timepoints (Fig. 5d), including biological processes that involved chemical and synaptic signaling and neuronal development. GO analysis was not conducted for cluster 6 as not enough genes were enriched at DIV31 to compare to DIV14, suggesting no significant change in biological processes with time in culture. Collectively, transcriptome analysis of the neurons in our complex culture system (relative to the simple system) suggests that the two neuronal clusters are associated with the maturation state of the cell in long term culture.

**Figure 6:**
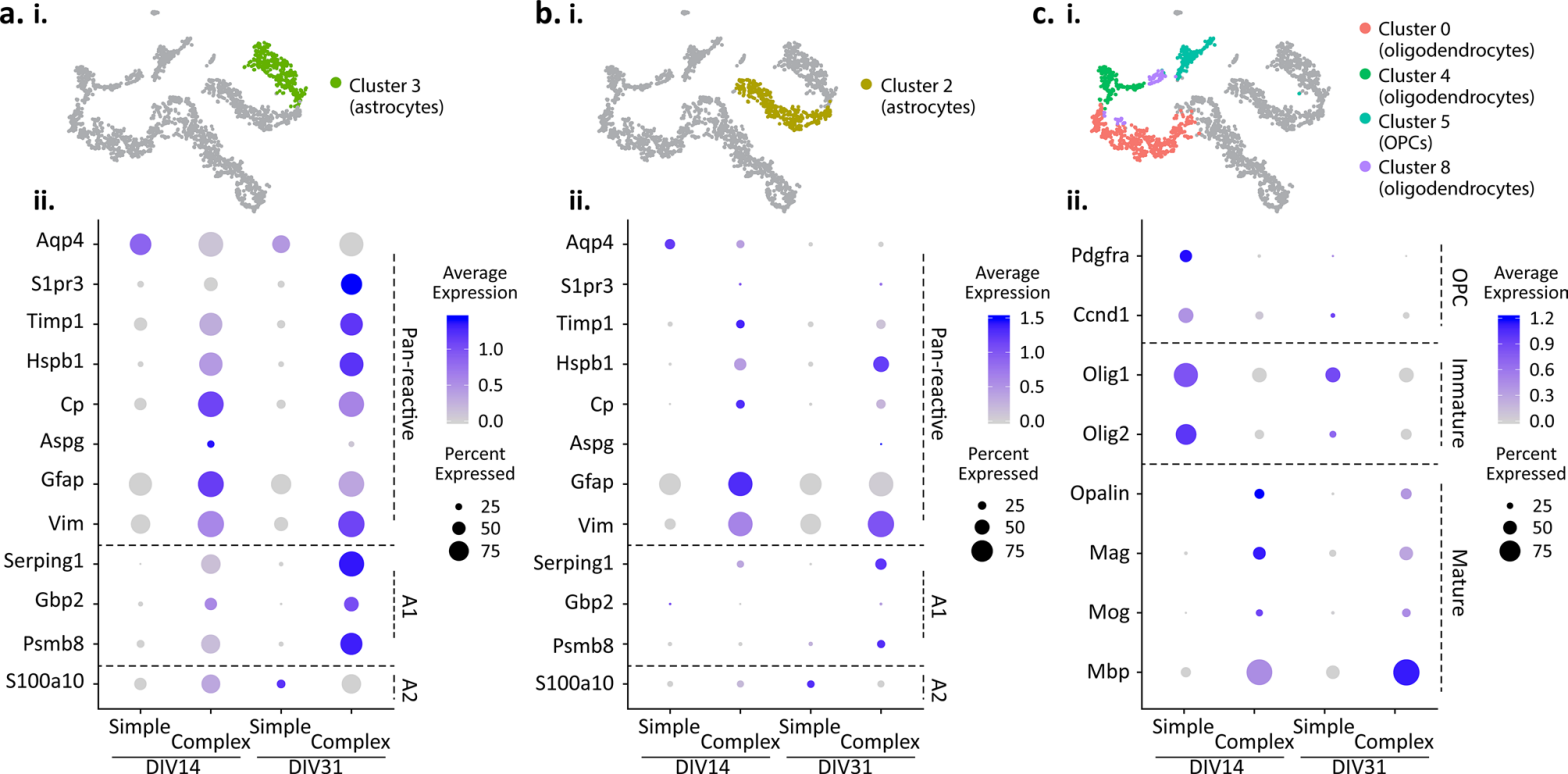
Characterization of astrocytes and oligodendrocytes. Dot plots represent key cell type marker gene expression within simple and complex cultures at DIV14 and DIV31. (**a**, **i**) t-SNE plot illustrating cluster 3 of astrocytes; (**a**, **ii**) Dot plot of cluster 3 astrocytes. (**b**, **i**) t-SNE plot illustrating cluster 2 of astrocytes. (**b**, **ii**) Dot plot of cluster 2 astrocytes (**c**, **i**) t-SNE plot illustrating clusters of oligodendrocytes. (**c**, **ii**) Dot plot of oligodendrocytes. Dot size indicates proportion of cells in cluster that express a gene; the shading indicates the average level of expression (low to high indicated as light to dark purple).

The astrocyte transcriptome in our simple and complex cultures displayed two subpopulations that persisted with time in culture: one cluster of cells was highly enriched with *Aqp4* (cluster 3, Fig. 3f), whereas the second cluster had little to no *Aqp4* expression despite exhibiting *Gfap* enrichment (cluster 2, Fig. 3e). Closer analysis of the astrocyte clusters using a list of reactive astrocyte genes associated with neuroinflammation^41^ also confirmed two distinct subpopulations, specifically in the complex co-cultures but not in the simple cultures (Fig. 6a, b). In the complex cultures, the high *Aqp4*-expressing subpopulation (cluster 3, Fig. 6ai) expressed a number of pan-reactive astrocyte genes (e.g., *S1pr3, Timp1, Hspb1, Cp, Aspg, Vim*) and A1-reactive astrocyte genes (e.g., *Serping1*, *Gbp2, Psmb8*) (Fig. 6aii). Notably, expression of these genes increased longitudinally, both through elevation of the expression level within individual cells or through an increase in the number of cells expressing these genes within the population (Fig. 6aii). While cluster 3 exhibited detectable levels of *s100a10,* a gene associated with the A2-reactive phenotype, the transcriptome analysis suggested that this subpopulation predominantly associates with the A1-reactive astrocyte phenotype. In contrast, the second astrocyte subpopulation (cluster 2, Fig. 6bi) displayed a reduced degree of reactivity with low expression levels of the pan-reactive genes (with the exception of *Gfap* and *Vim*) as well as A1- and A2-reactive astrocyte genes (Fig. 6bii). Interestingly, while simple cultures contained some low level of astrocytes, they did not exhibit an up-regulation of reactive astrocyte genes. Together, these data provide evidence that the cellular complexity can increase the reactivity of astrocytes in the co-culture, which may be one factor that contributes to neural network activity.

Simple and complex cultures contain cells within the oligodendrocyte lineage, enriched with *Pdgfra* and/or *Mbp* (Fig. 3g, h), that were clustered into four cell subpopulations (Fig. 6ci). Distinct to the complex culture is the detection of mature oligodendrocytes in > 75% of oligodendrocyte lineage cells, with high expression levels of *Opalin*, *Mag*, *Mog* and *Mbp* at both DIV 14 and 31 (Fig. 6cii). *Mbp* expression, in particular, was shown to increase with time in culture (Fig. 6cii), consistent with the immunostaining characterization (Fig. 2). Conversely, precursor and immature markers (i.e. *Pdgfra* and *Olig1*, respectively) were observed within simple cultures (Fig. 6cii) at the respective time points. Collectively, transcriptome analysis of oligodendrocyte lineage cells shows that the oligodendrocyte precursor cells present during the culture seeding developed into mature oligodendrocytes in the complex cultures, whereas they remained in an immature state in the simple cultures.

Given that neither simple nor complex cultures were directly supplemented with microglia, it was not surprising that only a miniscule population of microglia was detected (Fig. 3a). Nevertheless, the complex cultures had sufficient microglia counts to allow a transcriptome analysis (n = 12 cells at 14 DIV and n = 18 cells at 31 DIV). We examined the expression levels of genes associated with homeostatic and disease-associated microglial (DAM) phenotypes^42, 43^. In general, microglia expressed a subset of homeostatic genes (e.g., *Tmsb4x, C1qb, C1qc, C1qa, Csts, Cst3 Csf1r, Ctsd* and *Hexb*) with only *Tmsb4x* showing a time dependent increase in RNA counts (Supplementary Fig. S6)*.* Genes associated with a stage 1 DAM phenotype were also detected (e.g., *Apoe, Fth1, B2m, Lyz2, Tyrobp, Ctsb, Gnas,* and *Actb*), with only *Apoe* showing a time-dependent increase. A few genes from the stage 2 DAM phenotype were detected, albeit at low levels (e.g., *Trem2, Ctsl, Timp2*). These results suggest that the small subset of microglia within the complex culture condition identifies with both the homeostatic and stage 1 DAM phenotype. However, a more in depth study with a significantly greater population of microglia will be needed to determine whether (and how) this phenotype affects the dynamics of the complex culture.

### Electrophysiology

**Figure 7:**
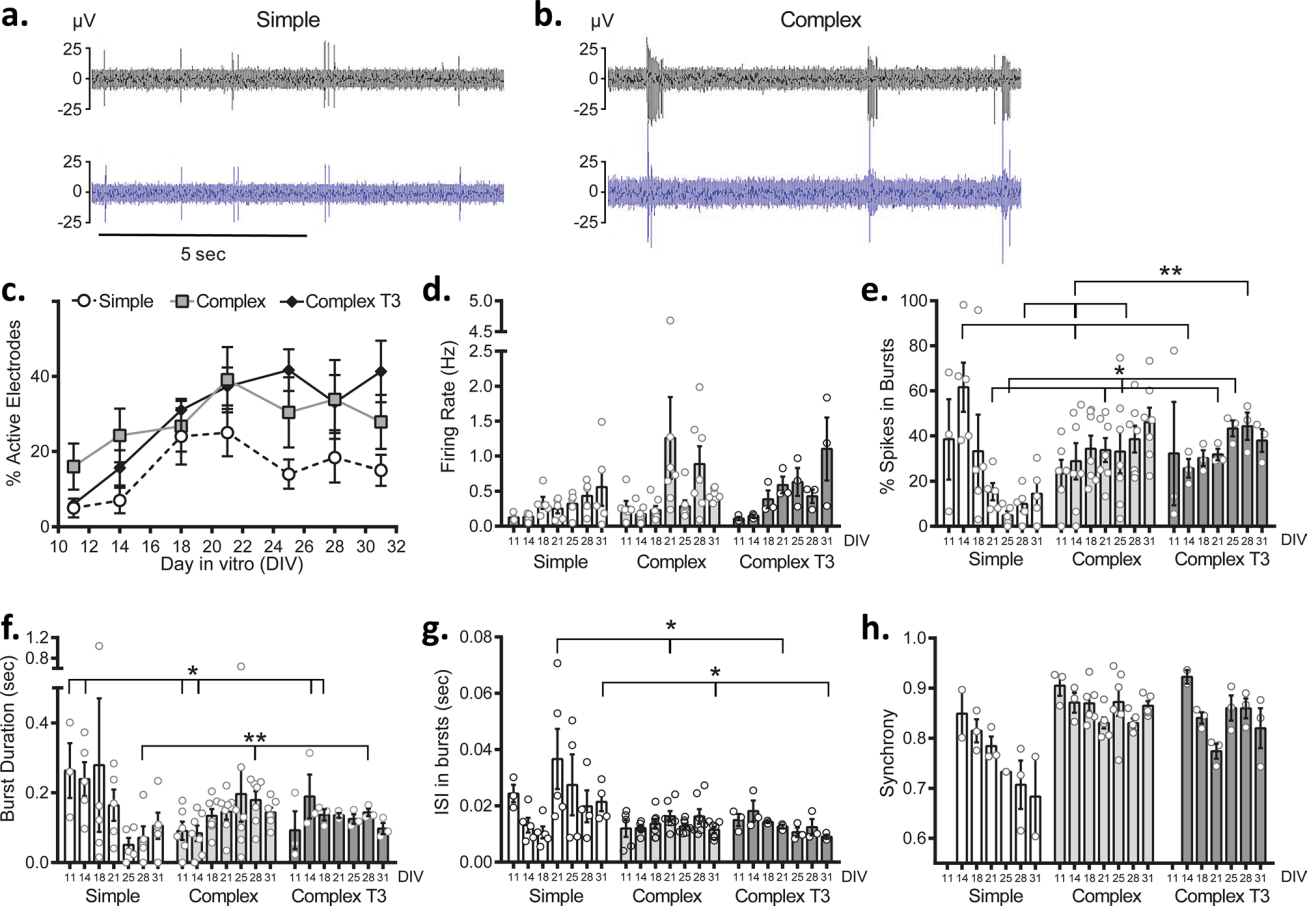
Baseline characteristics of simple and complex cultures. Two representative electrodes with neuronal activity are shown for simple and complex recordings in (**a**) and (**b**) at DIV31, respectively. Comparisons of activity and firing features over days in vitro (DIV) are shown including: (**c**), % active electrodes, (**d**), firing rate, (**e**), % spikes in bursts, (**f**), burst duration, (**g**), interspike interval within bursts (ISI) and (**h**), synchrony. Data are shown as mean ± SEM. Asterisks (*) indicate significance in two-way ANOVA. *p < 0.05, **p < 0.01. Asterisks associated with DIV indicate significance for culture type. Asterisks alone indicate significance for media type.

We compared spontaneous (baseline) neuronal electrophysiology between experimental groups over 31 days in culture. Examples of raw data traces for both simple and complex groups at DIV31 are shown in Fig. 7a, b, respectively. Relative to simple cultures, the complex cultures exhibited firing events that were primarily organized in distinct bursts (evident in Fig. 7b) as opposed to single spike activity. Representative raster plots, summarizing overall activity over the recording period, illustrate the progression of spiking activity for each culture system (simple and complex) from DIV14 to DIV31 (Supplementary Fig. S7). At DIV14, all systems showed spiking activity, although more distinct bursting was noted for both complex systems. By DIV31, spiking activity had increased for both systems; notable coordinated bursting activity was observed for both complex systems. In the simple system, spiking activity was generally more spurious and uncoordinated. Overall firing, burst duration, interspike interval in bursts, percent of spikes in bursts, and synchrony features typically reported for in vitro systems were compared between simple and complex systems. In general, complex networks were more active across DIV, as evidenced by a greater number of active electrodes (Fig. 7c) (~ 40% complex T3, ~ 27% complex, 15% simple at DIV31). While no significant changes in firing rate were observed between simple and complex groups (Fig. 7d), more spikes were clustered in bursts (Fig. 7e, DIV31) for complex (46.08% ± 6.47) and complex T3 systems (37.96% ± 5.09) than in the simple system (14.45% ± 5.18). Significant changes in firing rate were observed when comparing complex systems at a few time points (DIV18, p < 0.05; DIV25, p < 0.01; DIV31, p < 0.05). When evaluating specific bursting features, the burst duration (Fig. 7f) and the interspike interval within bursts (Fig. 7g) were consistent over time for both complex systems demonstrating earlier network maturity and stability of the complex network once formed. Additionally, both complex and complex T3 groups showed greater degrees of synchrony than the simple cultures (Fig. 3h; a value closer to 1 indicates higher synchronization). At DIV31, simple cultures were less synchronized (0.68 ± 0.08) when compared to both complex systems (complex, 0.87 ± 0.10, p < 0.05; complex T3, 0.820 ± 0.04). Furthermore, complex cultures synchronized earlier than simple cultures (DIV11 versus DIV14, respectively) and a greater percentage of devices demonstrated synchronicity when compared to the simple systems (Supplementary Fig. S8).

**Figure 8:**
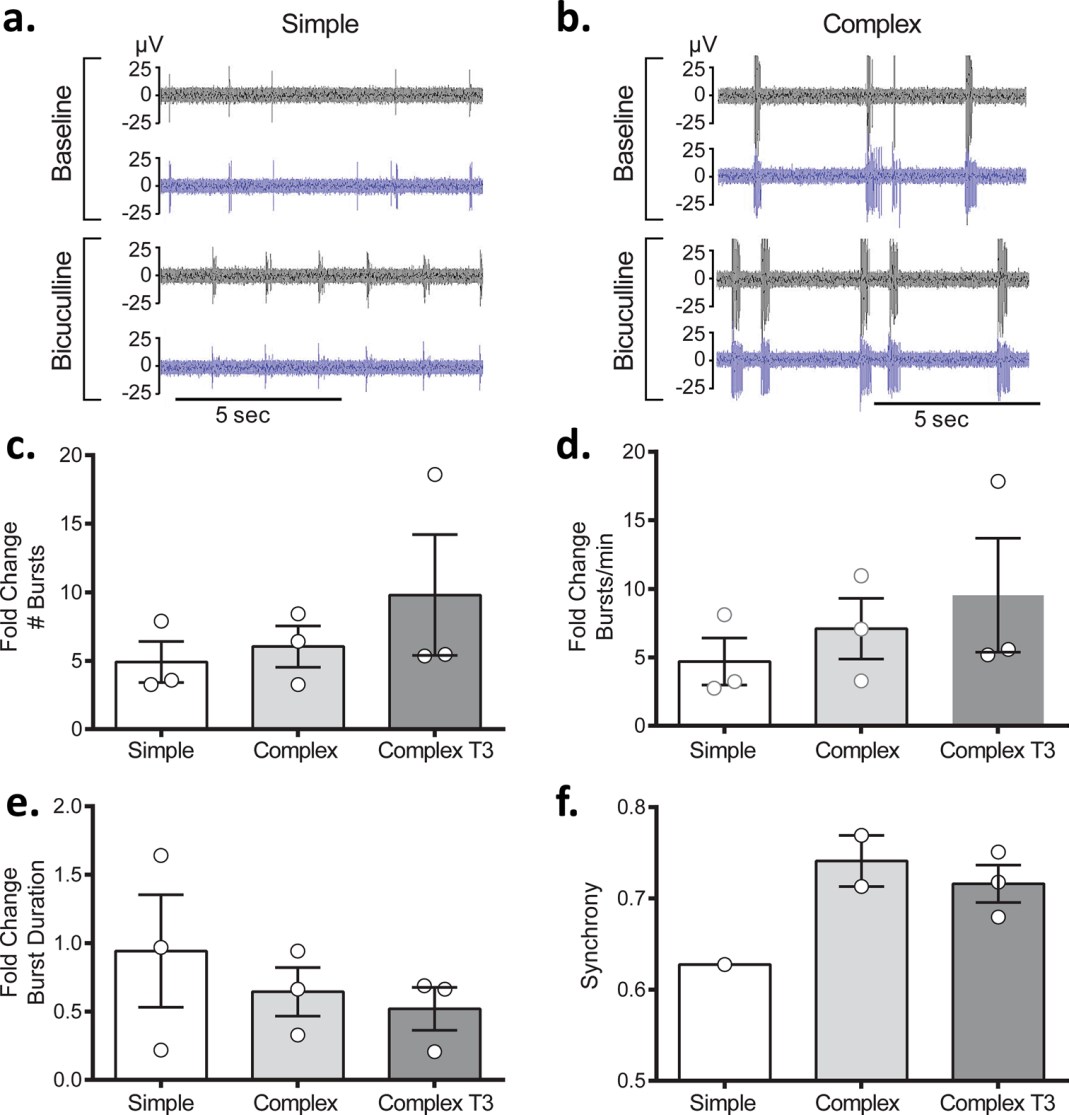
Responses of simple and complex cultures to bicuculline (BIC) at DIV32. Two representative electrodes for baseline and bicuculline evoked activity are shown for simple and complex recordings in (**a**) and (**b**), respectively. Comparisons of changes in specific features upon BIC exposure expressed as the fold change are shown, including: (**c**) # bursts, (**d**) bursts/min, (**e**) burst duration and (**f**) synchrony. Data are shown as mean ± SEM.

Next, we investigated the effects of the GABA antagonist bicuculline (BIC) to identify how the presence of the glial support cells affected functional responsiveness between the simple and complex systems. For both simple and complex cultures, the addition of bicuculline elicited the expected distinct, coordinated bursting behavior (Fig. 8a, b). However, a greater number of bursts (Fig. 8c) and increased burst rate (Fig. 8d) were noted for complex systems relative to simple, although these were not statistically significant. Complex cultures also demonstrated a slight decrease in burst duration (Fig. 8e) and greater synchronization upon addition of BIC (Fig. 8f). Only 33% of simple cultures showed synchronous activity compared to 67% and 100% for the complex and complex T3 systems, respectively.

## Discussion

Our study aimed to identify the morphological, molecular, and functional differences between a simple neuronal culture and a co-culture system comprised of neurons, astrocytes, and oligodendrocytes seeded in ratios relevant to the postnatal brain^27–29^. Thus, we hypothesized that by increasing the cellular complexity of in vitro systems, we could more closely recapitulate in vivo physiology and function. This is the first report that has characterized a neuron and glia co-culture system with neurons, astrocytes, and oligodendrocytes on an MEA that directly correlates neuronal network functionality with cellular and molecular composition. In our complex system, we have demonstrated: (1) notable differences in the network arrangement and morphology, with highly localized regions of neuronal density and distinct synaptophysin expression, (2) myelin production and axonal co-localization in complex systems supplemented with T3, (3) higher expression for genes involved in neuronal synapse and network formation as well as cell signaling processes and (4) distinct electrophysiological activity, exemplified by accelerated network maturation, higher degrees of synchronization, and overall more neuronal activity.

The intricate functionality of the central nervous system is contingent on not only neurons but also glial cells, including astrocytes and oligodendrocytes. Inclusion of these cell types is key in recapitulating functional aspects of the brain in an in vitro system. Astrocytes and oligodendrocytes play a critical role in neuronal maturation and function; processes such as synaptogenesis, neuritogenesis, and metabolic support are tightly coupled to the interaction with these glial cell and their secreted growth factors^32, 35, 44–46^. Astrocytes alone have been shown to influence network formation, whereby secreted factors such as lipids and extracellular matrix molecules (e.g., thrombospondins and glypicans) have been shown to promote neuron outgrowth and synapse formation^47, 48^. The myelination of axons early in development, mediated by mature oligodendrocytes, is also important for proper neuronal function. In addition to contributing to axonal health and stability^49^, mature oligodendrocytes have the role of generating the myelin sheaths around axons in the central nervous system; this myelination enhances speed and efficacy of nerve pulse conduction^50^.

To elucidate the effects of neuroglia on overall culture morphology and neuronal function, we characterized our simple and complex cultures using immunostaining, single-cell RNA sequencing, and electrophysiology over 31 days in culture. Despite the low levels of contaminating astrocytes and oligodendrocytes present in the simple neuron cultures, complex cultures with defined cell-type ratios exhibited distinct morphology (Figs. 1, 2), higher expression of complex neuronal processes (Figs. 4, 5), and well-defined electrical activity (Fig. 7) relative to the simple cultures. Using scRNA-seq, our complex cultures revealed subtypes of the neurons, astrocytes and oligodendrocyte precursor cells initially seeded. (Fig. 3). For instance, neurons displayed two cell clusters (i.e., 1 and 6) that were associated with the maturation state of the neurons (i.e., immature and mature, Fig. 4), which was dependent on the time in culture (Fig. 5). At DIV14, complex cultures exhibited a higher expression of genes, relative to simple cultures, associated with neuronal network and synapse formation (Fig. 5c), while at DIV31 a shift in biological processes towards cell signaling, homeostasis, and responsiveness was observed in these complex cultures (Fig. 5e). In-depth characterization of the non-neuronal cells within our complex system revealed a greater number of mature oligodendrocytes at DIV14, compared to the immature cell population in age-matched simple cultures, and remained in their mature state for the duration of the long-term culture (Fig. 6c). Astrocytes, however, displayed two cell clusters that were associated with the reactive phenotype described in neuroinflammation in vivo and induced in vitro via exogenous application of cytokines^41^. Notably, this reactive phenotype was only observed in our complex cultures, suggesting that the interaction between the different cell types can induce the pan-reactive- and A1 neurotoxic-like phenotypes observed in vitro (Fig. 6a, b, respectively). In particular, these characteristics illustrate that cellular complexity within these systems significantly influences cell-specific phenotypes, which may have influenced neural network activity. Indeed, functional differences noted for our complex system, including earlier spiking activity and coordinated bursting, suggest a relationship between neural network activity and phenotypic changes of the seeded glial cells that was not observed in simple cultures. In addition to molecular (scRNA-seq) findings, we observed structural features distinct to complex cultures, including synaptophysin staining in dense neuronal regions (Supplementary Fig. S3) and co-localization of myelin basic protein (MBP) with mature axons (p-NF-H) in the complex T3 group (Fig. 2). Others have demonstrated myelination in cultures of dissociated CNS and spinal neurons, which is enhanced by supplementing the culture media with triiodothyronine (T3) for increasing alignment of MBP with mature axons^36^. In the work by Pang et al.^36^, neuron-oligodendrocyte rat embryonic cortical co-cultures were established using T3 in their media; myelination was shown to peak around 6 weeks (DIV40) and nodes of Ranvier were identified using caspase reactivity. Here, we chose to evaluate if the addition of T3 would further enhance our complex system by eliciting similar structural (MBP/axonal alignment) changes, and to ultimately correlate this with possible accelerated network formation and function. While structural differences between these two systems were noted (Fig. 2h, l), functionally, only two firing features (firing rate and interspike interval within bursts) were found to be statistically different between these complex systems (Fig. 7d, g). This observation suggests that the majority of functional changes observed for our complex system can be attributed to the presence of astrocytes and oligodendrocytes in our cultures, rather than MBP alignment on mature axons within our cultures. However, culture time (DIV31 compared to DIV40 for Pang et al.^36^) and a smaller group size for our complex T3 group (n = 3) could have influenced these data, and we posit that more significant differences may be apparent at later time points. Collectively, reactive astrocytes and oligodendrocyte-lineage cells were present in complex cultures that exhibited distinct changes in neural network activity (relative to simple cultures). It will be important for future studies to identify how the phenotypes of these glial cells contribute to neural network maturation and modulation in the complex co-cultures.

Finally, we evaluated whether cell composition dictated the responsiveness of simple and complex systems to the GABA_A_ receptor blocker, bicuculline (BIC). BIC antagonizes GABA-mediated inhibition which results in periodic bursting activity or convulsions when administered in vivo^51, 52^. BIC is frequently used to evaluate neuronal composition (i.e., evaluate the presence of GABAergic neurons) and network behavior of in vitro systems^23, 53^. While not statistically significant, the complex systems were more responsive to BIC when compared to their baseline activity, as demonstrated by the fold-change increase in number of bursts and for burst rate (Fig. 8), suggesting more convulsion-like behavior for the complex systems. This could be due to direct effects of BIC on the glial cells, as GABA_A_ receptor expression has been shown in both astrocytes^54, 55^ and oligodendrocytes^56^. Alternatively, BIC may elicit release of gliotransmitters by astrocytes (i.e. glutamate, cytokines and GABA) to regulate downstream neuronal function. Gliotransmitters, while similar to neurotransmitters, have been shown to modulate synaptic activity and network excitability, through different mechanisms^57, 58^. While further investigation is required to identify the mechanism of action, the differential responses observed between systems may be attributed to the differences in cellular composition. Ultimately, the inclusion of astrocytes and oligodendrocytes and their role in regulating and influencing neuronal function has enhanced neuronal responsiveness in our complex systems.

Taken together, our results demonstrate that there are distinct morphological, molecular, and functional differences between our simple and complex in vitro systems. By evaluating neurons as a part of a more complex (and relevant) physiological system, this co-culture system provides a test bed that is one step closer to mimicking the in vivo environment, and may provide additional insight when evaluating drugs, toxicants, and disease mechanisms by relying not solely on the immediate effects of these conditions on neuronal activity, but by also capturing secondary effects on the support cell ecosystem. Our study represents only a single step towards enhancing the physiological and functional relevance of our neuronal cultures, as additional modifications, including the introduction of relevant levels of microglia, supplementing cultures with extracellular matrix, and incorporating three-dimensional architecture, will add additional levels of complexity and may ultimately bring us closer to recapitulating in vivo function. Future work will expand the characterization of additional chemicals to evaluate whether (and to what degree) our complex system more closely mimics the neuronal responses observed in vivo. Furthermore, this approach can also be extended to create a more relevant human culture system by incorporating human-derived neurons and neuroglia, which will be important for evaluating human-relevant outcomes.

## Methods

### Chemicals

Primary Neuron Basal Medium (PNBM) and all supplements were purchased from Lonza (Walkersville, MD, USA). Triiodothyronine (T3) and bicuculline were purchased from Sigma Aldrich (St. Louis, MO, USA).

### Microelectrode array (MEA) fabrication

The MEA device was microfabricated as previously described^19, 26^. Briefly, MEAs (50 µm platinum electrodes) were fabricated on a glass substrate. Standard photolithography and wet or plasma etching were used to pattern metal constituting the 60 electrodes and electrode traces; polyimide was deposited and patterned as an insulating layer over the metal traces. A polystyrene cylinder was affixed over the MEA to enable cell culturing over the array. The well area was 113 mm^2^ and accommodated approximately 700 μL. ZIF connectors were added to the device for electrical connections and electrodes were plated with platinum black^53^. Impedance measurements were taken prior to seeding and ranged from ~ 50 to 250 kΩ at 1 kHz.

### Cell seeding

MEAs were sterilized with 70% ethanol for 20 min, rinsed 5 × with sterile water, and coated with 0.1 mg/mL poly-d-lysine in borate buffer prior to seeding. Primary rat embryonic cortical neurons (Lonza, Walkersville, MD, USA) were seeded at a density of 135 K cells/device (n = 5 for simple, n = 7 for complex, n = 3 for complex T3). Two days after neuron seeding, primary rat postnatal astrocytes (27 K/device, Lonza, Walkersville, MD, USA) and oligodendrocyte precursor cells (9 K/device, Sciencell, Carlsbad, CA, USA) were added to neuronal cultures for the co-culture groups. Additional wells for each culture system were seeded in 96 well plates (38 K neurons, 7.6 K astrocytes, 2.6 K oligodendrocytes) for immunocytochemistry and single cell RNA-sequencing characterization. Cell ratios (~ 79% neurons, ~ 16%, ~ 5%) were informed by reports for these cell types during postnatal periods^27–29^. Cultures were maintained with twice-weekly exchanges of 50% media [Primary Neuron Basal Medium (PNBM) supplemented with 2 mM l-glutamine, 50 μg/mL gentamicin, 37 ng/mL amphotericin, and 2% NSF-1] in a humidified incubator (5% CO_2_, 37 °C). For the complex T3 group, T3 (60 ng/mL) was added to the media starting at DIV10. Custom device caps, made from a polytetrafluoroethylene (PTFE) housing and a fluorinated ethylene–propylene (FEP) membrane, were used to maintain sterility and to allow for gas exchange^59^.

### Immunocytochemistry

Cells were rinsed 4× with 1× PBS, fixed with 4% paraformaldehyde, washed with PBS (4×), and permeabilized using 10% saponin before blocking with 10% goat serum (1 h at room temperature). Primary antibodies used included: class III beta-tubulin for neurons (Tuj-1, chicken, Neuromics, Edina, MN, USA, 1:200 dilution), glial fibrillary acidic protein (GFAP) for astrocytes (rabbit, Millipore, Burlington, MA, USA, 1:1,000), myelin basic protein (MBP) for mature oligodendrocytes and myelin (mouse, Millipore, Burlington, MA, USA, 1:1,000) and pre-synaptic marker synaptophysin (rabbit, Abcam, Cambridge, MA, USA, 1:250). After primary antibody incubation (overnight at 4 °C), cells were washed with PBS (4×) and stained with secondary antibodies (1 h at 37 °C). Secondary antibodies included: goat anti-mouse linked to Alexa Fluor (AF) 488, goat anti-chicken linked to Alexa Fluor 647 and goat anti-rabbit linked to Alexa Fluor 594 (1:500 dilution, Life Technologies, Eugene, OR, USA). AF-594 conjugated phosphorylated neurofilament H was used to stain mature axons (p-NF-H, Biolegend, San Diego, CA, USA). After secondary antibody incubation, the cells were washed with PBS (4×), and incubated (10 min) with the nuclear stain, diamidino-2-phenylindole (DAPI, ThermoFisher, 300 nM), before imaging. A LSM700 confocal microscope (Carl Zeiss Microscopy, Thornwood, NY, USA) was used for imaging. For MBP quantification, a threshold was set using image J across images from all groups. Percent of MBP/total image area was calculated and averaged from three to five fields of view of each culture type (n = 3). For neuron quantification at DIV31, tuj-1^+^ cells were manually counted and represented as the fraction of total DAPI positive cells. DAPI counts were determined using ImageJ. Three to four fields of view were quantified for each well analyzed (n = 3/group).

### Single-cell RNA-sequencing data generation

At each time point (DIV14 and DIV31) cultured cells were detached with 0.1% trypsin–EDTA, washed and triturated using a fire-polished Pasteur pipette to a single cell suspension in 10% FBS in PBS. Cells were pelleted at 200 rcf for 5 min at 4 °C and washed with PBS to remove residual FBS. Final cell counts were performed using an automated cell counter (Countess, Thermo Fisher Scientific, Waltham, MA, USA) and cells were resuspended in PBS + 0.04% nonacetylated BSA before single cell suspension generation using a Chromium Controller (10 × Genomics, Pleasanton, CA, USA) following manufaturer’s recommendations using the Chromium Single Cell 3′GEM, Library and Gel Bead Kit v3 (10 × Genomics, Pleasanton, CA, USA). Sequencing libraries were qualitatively analyzed using an 2,100 Bioanalyzer (Agilent, Santa Clara, CA, USA) for library size, quantitated using Qubit 2.0 (Thermo Fisher Scientific, Waltham, MA, USA), and subsequently sequenced on a NextSeq 500 (Illumina, San Diego, CA, USA).

### Single-cell RNA-sequencing data processing and analyses

Single-cell RNA sequencing data analysis was performed using the Cell Ranger Single-Cell Software Suite (10× Genomics, Pleasanton, CA, USA) and Seurat^60^. Cell Ranger was used to perform sample demultiplexing, barcode processing, and 3′gene counting. Samples were aligned to the rat genome (rn6) using “cellranger mkfastq” with default parameters. Unique molecular identifier (UMI) counts were generated using “cellranger count”. Subsequently, an integrated analysis of samples from four experimental groups was performed using Seurat to identify and compare various cell types. First, cells with fewer than 200 detected genes per cell and genes that were expressed by fewer than five cells were filtered out. Prior to integrating the datasets, a log-normalization was performed using ‘NormalizeData’ function implemented in Seurat. Subsequently, the 2000 most variable genes in each dataset were identified using ‘FindVariableFeatures’ function. Next, integration anchors were identified using ‘FindIntegrationAnchors’ function with default parameters and all four datasets were integrated using ‘IntegrateData’ to generate a new integrated matrix. The integrated matrix was then scaled to a mean of 0 and variance of 1 and the dimensionality of the data was reduced by principal component analysis (PCA) using the variable genes. Then, a graph-based clustering approach was used to cluster cells. A K-nearest-neighbor (KNN) graph was constructed using the “FindNeighbors” function with default parameters and then the Louvain algorithm was used to iteratively group cells together by “FindClusters” function (resolution = 0.2). Subsequently, the results were visualized using T-distributed Stochastic Neighbor Embedding (t-SNE) plots.

Gene ontology (GO) biological processes enrichment analysis was conducted for cell clusters and time points of interest using the tool, ToppGene (https://toppgene.cchmc.org/prioritization.jsp) ^61^.

### Chemical dosing

Bicuculline was prepared in primary neuron basal media (PNBM) to 4× the final desired in vitro concentration. For chemical exposures, 25% of the culture media was removed from the MEA well and replaced with an equal volume of 4× chemical working stock, resulting in a final concentration of 10 μM.

### Electrophysiology recording and processing

For electrophysiology measurements, devices were placed on a heated stage (37 °C) in a 5% CO_2_ controlled chamber during recordings. Electrophysiology measurements were acquired using a multi-channel recording system (AlphaLab SnR, Alpha Omega, Alpharetta, GA, USA) and were sampled at a frequency of 22.3 kHz and bandpass filtered between 268 and 8,036 Hz. Baseline and chemical exposure measurements were recorded for 10 min.

### Feature analysis and statistics

Feature analysis was carried out with a custom R package developed in-house. Spikes were detected using a threshold detection at − 4.5× the standard deviation of the noise from each channel. Heater spikes generated by the temperature controller used in this study were removed prior to analysis, and were identified using the following procedure: using a sliding window of 1 ms, spikes with voltage below − 200 μV that affected at least 80% of all channels in the device were considered heater spikes. Due to the fact that these heater spikes cause subsequent waves in the raw voltage that can be detected as spikes, all spikes within 6 ms of the heater spikes were removed as well. After heater spike removal, channels with fewer than ten spikes were removed from analysis. Bursts were defined as having a maximum beginning interspike interval of 0.1 s, a maximum end interspike interval of 0.2 s, a minimum interburst interval of 0.5 s, a minimum burst duration of 0.05 s, and a minimum of six spikes per burst^19^. Features were calculated per channel for each device. Mean values per device were calculated for each feature. Calculated features included: overall firing rate, burst duration, interspike interval within bursts and percent of spikes outside of bursts. These features have been previously described^4^. Additionally, we computed electrode synchrony. Synchrony was measured using the SPIKE distance^62^, which was computed with the *PySpike* package^63^. The SPIKE-distance between every pair of active electrodes was computed for each MEA. Since two spike trains with uniformly random activity may exhibit relatively high synchrony merely due to randomness, the SPIKE-distance from recorded data was normalized to random samples generated computationally. That is, for a pair of electrodes with n1 and n2 spikes, random spike trains were generated by sampling n1 and n2 points uniformly at random in a 10-min timeline. This process was repeated 1,000 times, and the average normalized score is reported. The SPIKE distance is a measure of *dissimilarity*, so, in order to report synchrony, the distance values obtained from *PySpike* were subtracted from 1. This way, synchrony values close to 1 denote a high degree of synchrony, and values close to 0 denote asynchrony. For drug challenges, the difference in electrophysiological activity before and after challenge was calculated and reported as fold change. A two-way analysis of variance (ANOVA) test followed by a Tukey honest significant difference post hoc test was used to analyze data across DIV. A one-way analysis of variance (ANOVA) test followed by a Tukey post hoc test was used to analyze %MBP expression (Fig. 2) and fold-changes for bicuculline data (Fig. 4). A p value of < 0.05 was considered significant. All error bars indicate standard error of the mean (SEM).

## Supplementary information

Supplementary file1 (CSV 3737 kb)

Supplementary file2 (PDF 1748 kb)

## Data Availability

The analysis code used to analyze data in this study is available upon request.
