# Supplementary material for: Functional and transcriptional characterization of complex neuronal co-cultures
Source: Sci Rep. 2020 Jul 3;10:11007. doi: 10.1038/s41598-020-67691-2 (PMC7335084; doi:10.1038/s41598-020-67691-2)
Supplement: Supplementary file 2 — Supplementary file2 (PDF 1748 kb) [file 41598_2020_67691_MOESM2_ESM.pdf]

# Functional and transcriptional characterization of complex neuronal co-cultures

Heather A. Enright[1,*], Doris Lam[1], Aimy Sebastian[1], Ana Paula Sales[2], Jose Cadena[2], Nicholas R. Hum[1], Joanne J. Osburn[1], Sandra K. G. Peters[1], Bryan Petkus[1], David A. Soscia[2], Kristen S. Kulp[1], Gabriela G. Loots[1], Elizabeth K. Wheeler[2], and Nicholas O. Fischer[1,*]

[1]Physical and Life Sciences Directorate, Lawrence Livermore National Laboratory, Livermore, CA, USA
[2]Engineering Directorate, Lawrence Livermore National Laboratory, Livermore, CA, USA

*email: enright3@llnl.gov, fischer29@llnl.gov

Supplementary Information

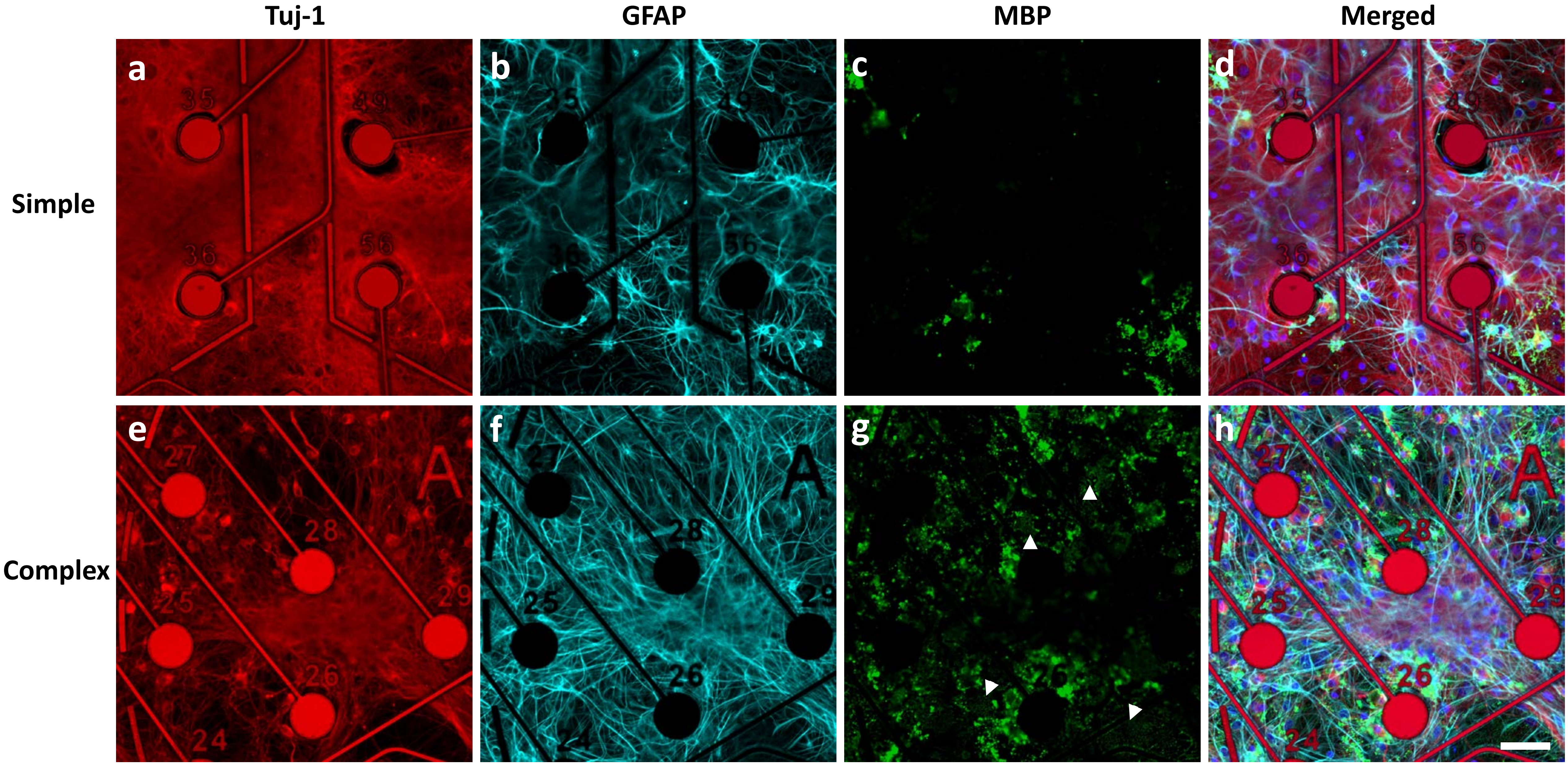


**Supplemental Fig 1. Original version of Fig 1 wherein electrode autofluorescence has not been removed from Tuj-1 channel.** Immunofluorescence characterization of cortical cultures in simple and complex systems at DIV31. Neurons were identified by staining for Tuj-1 (Neuron-specific class III beta-tubulin, **a, e**). Glial fibrillary acidic protein (GFAP) was used to identify astrocytes (**b, f**) and myelin basic protein (MBP) was used to identify mature oligodendrocytes and myelin (white arrowheads) (**c, g**). Merged images with nuclear stain (DAPI, blue) are shown in **d** and **h.** Scale bar = 50 µm

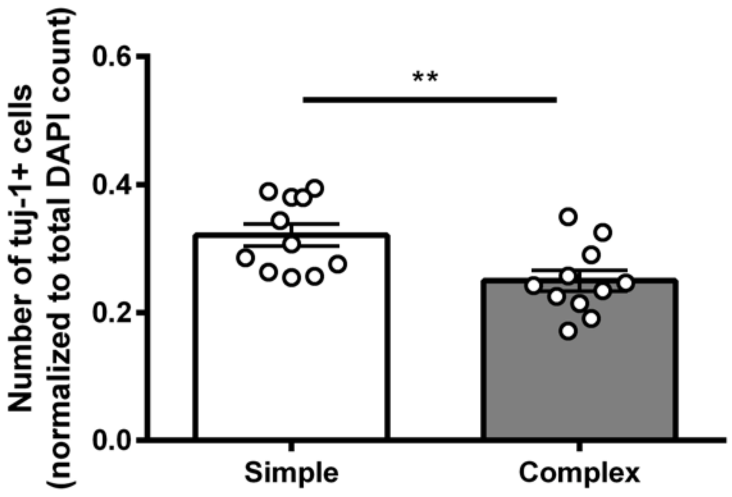


**Supplemental Fig 2.** Neuron counts for simple and complex groups at day in vitro (DIV) 31. Number of tuj-1+ cells are shown relative to total DAPI cell count (n=11 images/group across three individual wells). Data are shown as mean $\pm$ s.e.m. Asterisks (**) indicate significance in t-test. **$p<0.01$

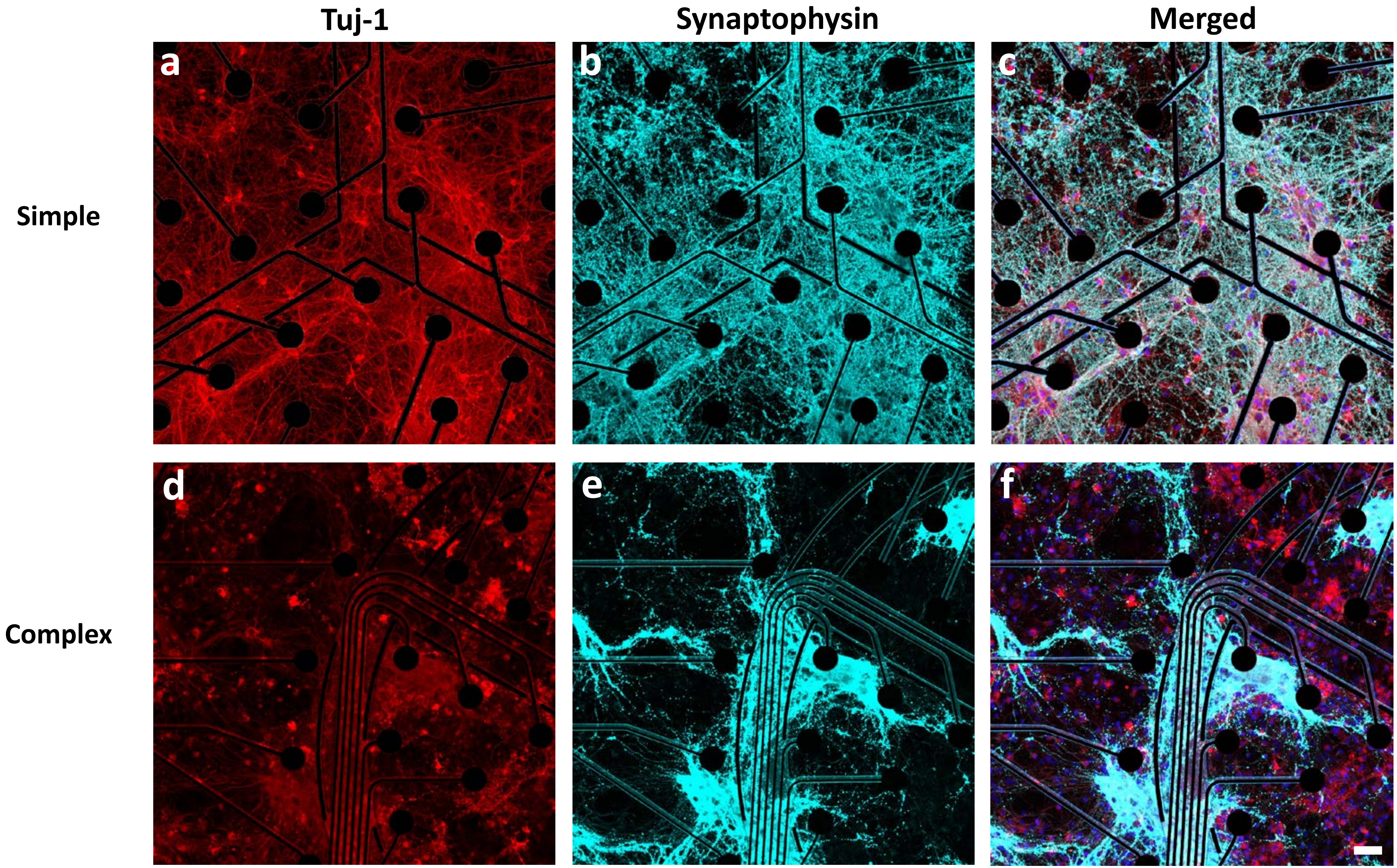


**Supplemental Fig 3.** Immunofluorescence characterization of network maturity in simple and complex systems at DIV31. Neurons were identified by staining for Tuj-1 (Neuron-specific class III beta-tubulin, **a, d**). Synaptophysin was used as a pre-synaptic marker (**b, e**). Merged images with nuclear stain (DAPI, blue) are shown in **c** and **f.** Figure has been modified to remove electrode autofluorescence. Scale bar = 50 µm

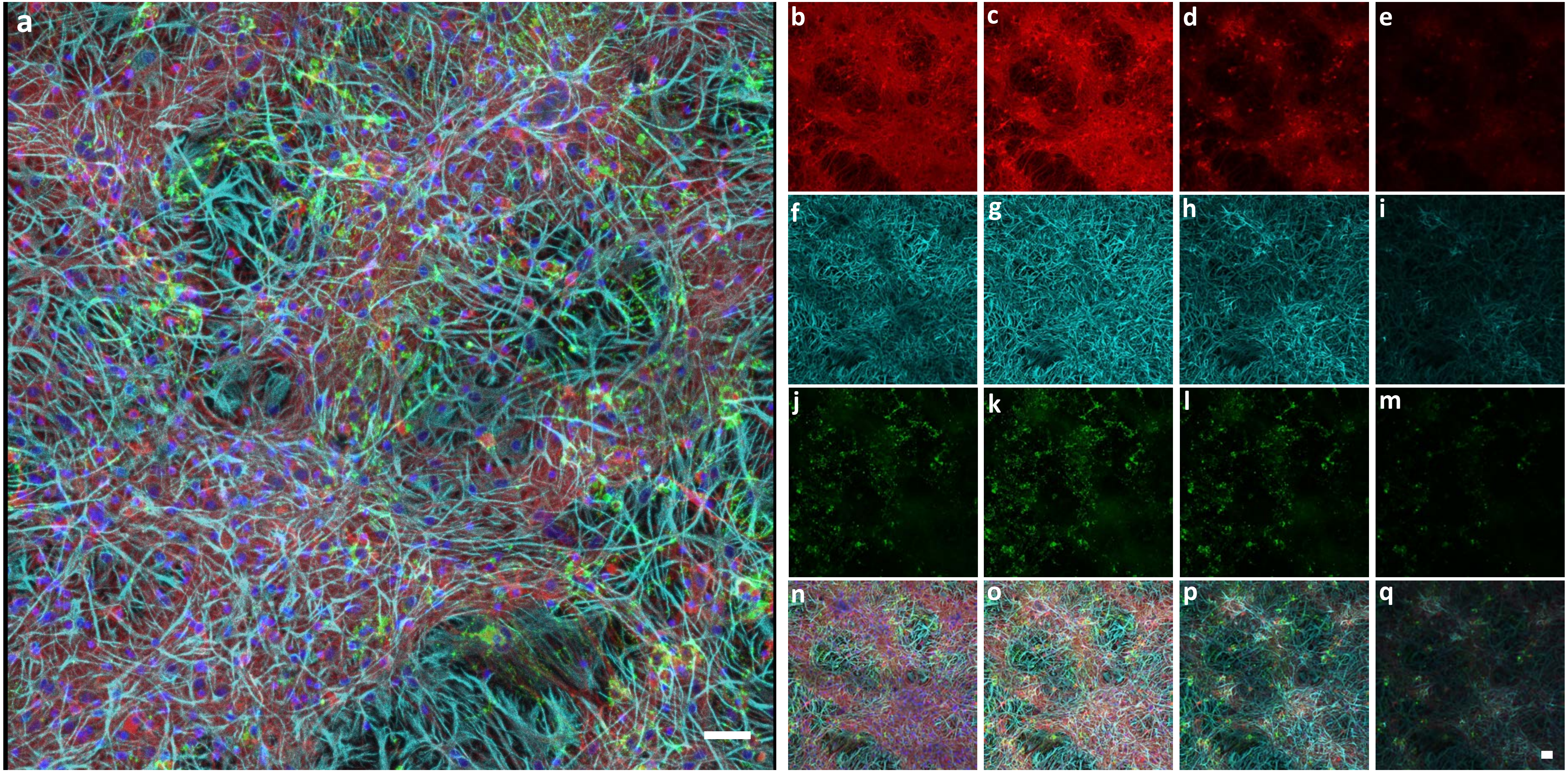


**Supplemental Fig 4.** Z-stack immunofluorescence characterization of cell types in a complex system at DIV31. Panel **a** shows a z-projection of all cell types (neurons = red, astrocytes = cyan, myelin basic protein (MBP) = green) and DAPI (blue). Neurons were identified by staining for tuj-1 (Neuron-specific class III beta-tubulin, **b-e**). Glial fibrillary acidic protein (GFAP) was used to identify astrocytes (**f-i**) and myelin basic protein (MBP) was used to identify mature oligodendrocytes and myelin (**j-m**). Merged images with DAPI are shown in **n-q**. Scale bars = 50 µm. Step size = 9 µm.

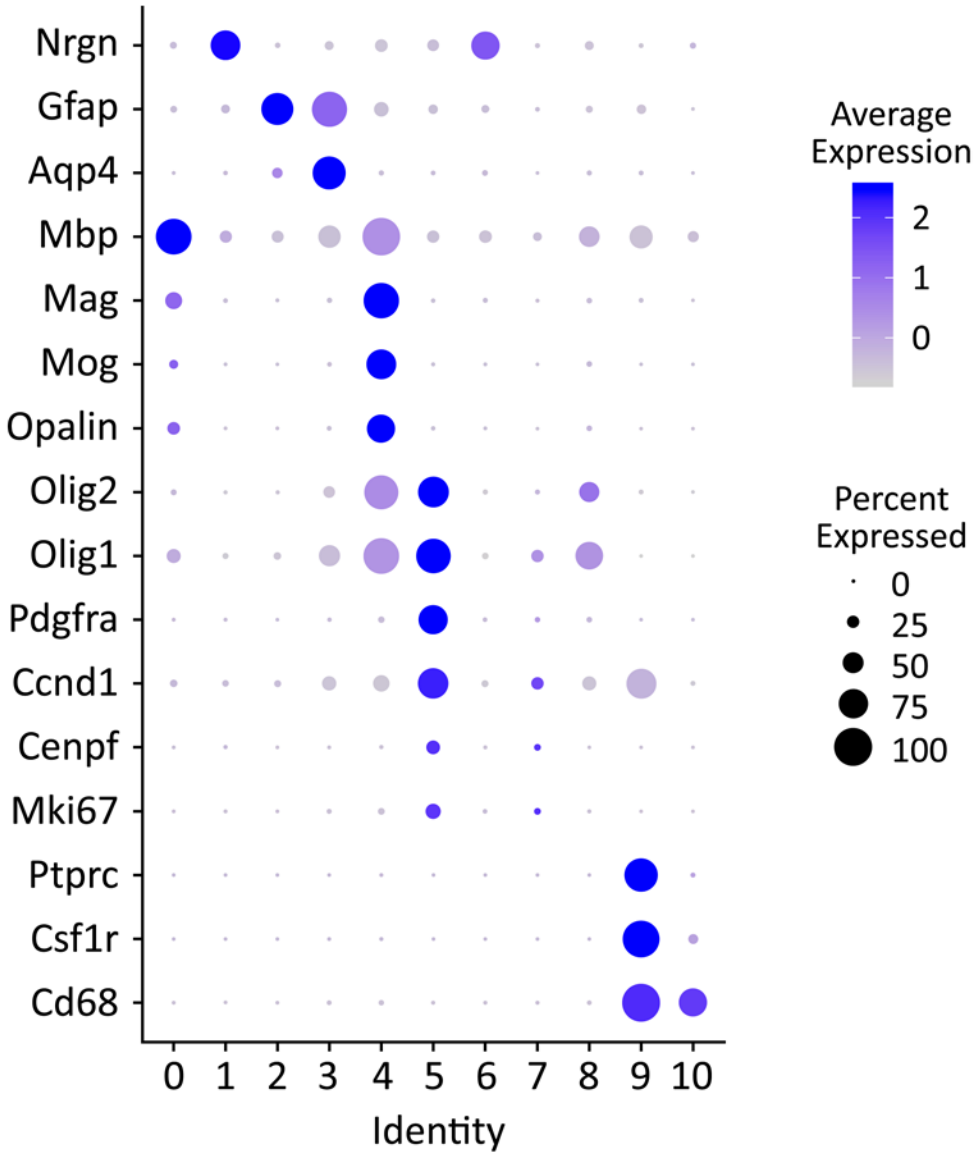


**Supplemental Fig 5.** Dot plot illustrating gene markers used to to identify each cluster. Dot size indicates proportion of cells in cluster that express a gene; the shading indicates the average level of expression (low to high indicated as light to dark purple).

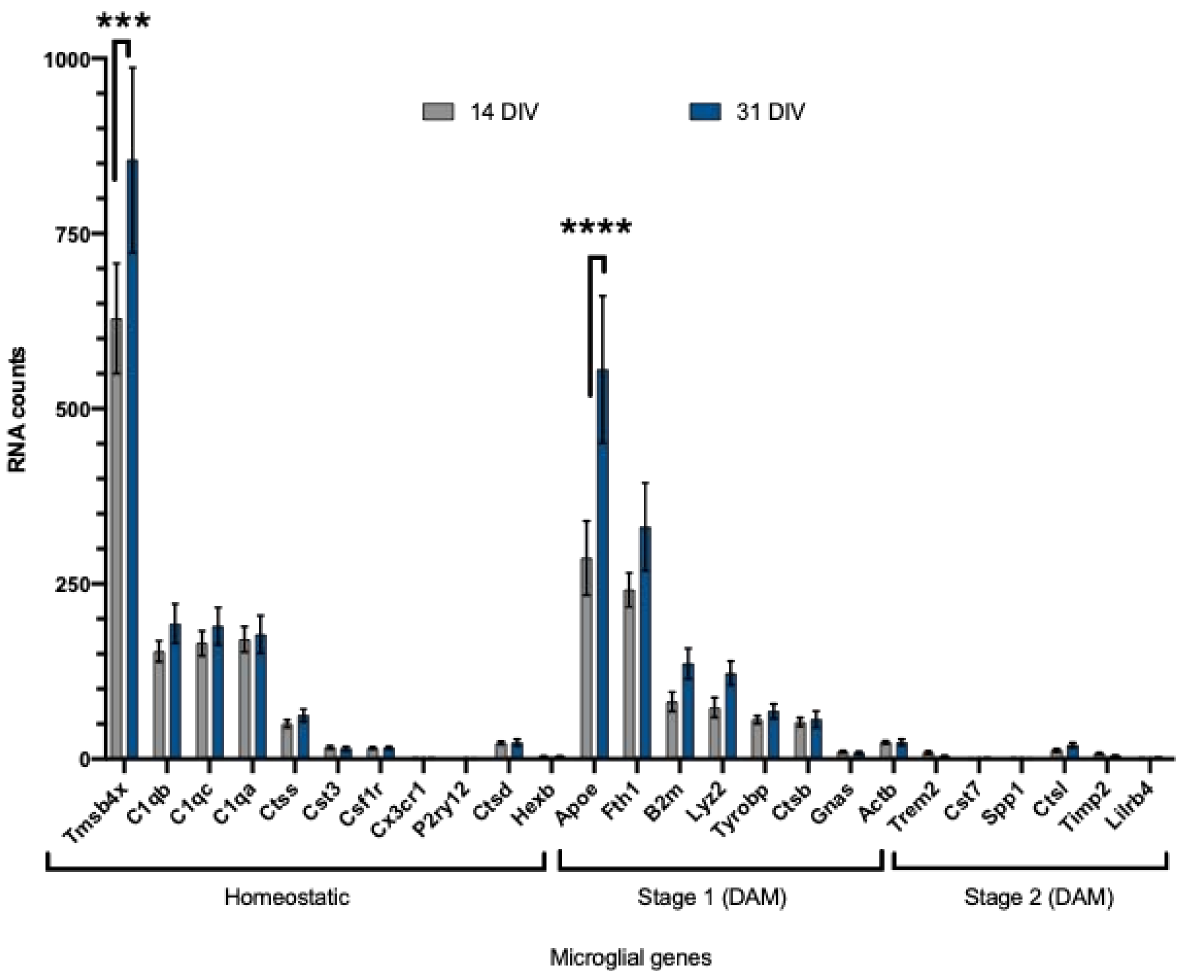


**Supplemental Fig 6.** Single-cell transcriptome analysis of microglia from complex cultures at 14 (grey) and 31 (blue) DIV. Bar graph shows the quantification of RNA counts for genes associated with homeostatic, and Stage 1 and Stage 2 Disease-associated microglia (DAM) phenotype. Data are shown as mean ± s.e.m. Asterisks (*) indicate significance between time points in two-way ANOVA followed by Sidak post-hoc test. ***p<0.001, ****p<0.0001.

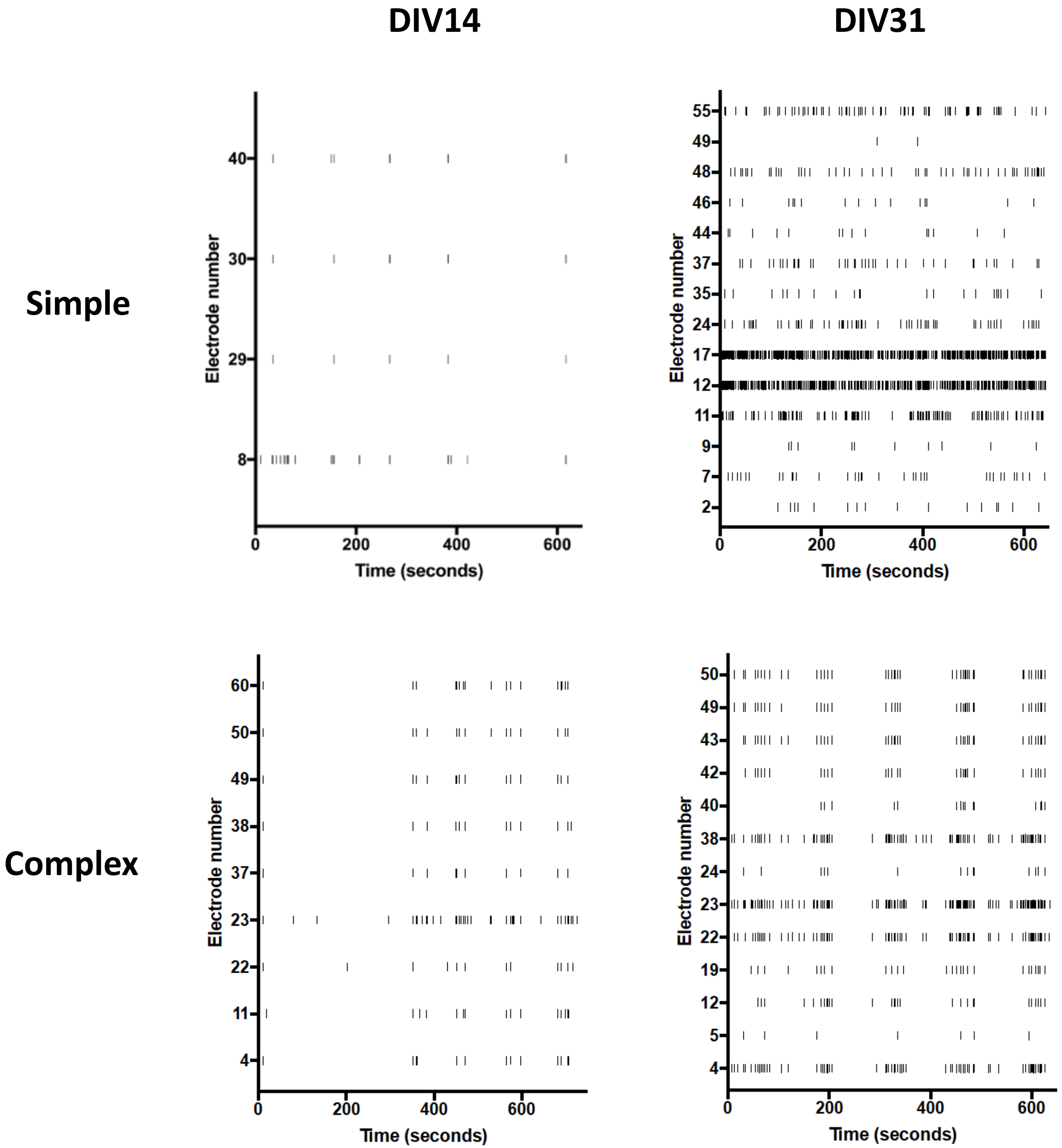


**Supplemental Fig 7.** Raster visualization of baseline activity in simple (top) and complex (bottom) cultures at DIV14 and DIV31. Each hash mark represents an action potential.

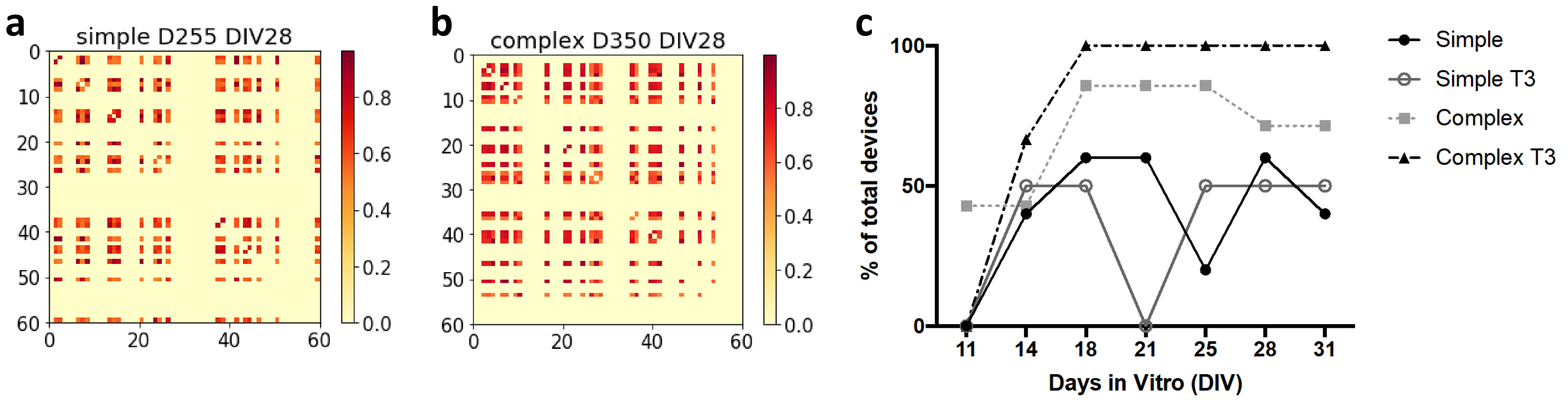


**Supplemental Fig 8.** Synchrony between simple and complex groups using cross-correlation. **a.** Examples of pairwise synchrony in simple and complex devices at DIV28. **b.** Percent of devices exhibiting synchronicity in simple and complex cultures over days in vitro (DIV).
